# Supplementary material for: Motivating participation in open science by examining researcher incentives
Source: eLife. 2017 Oct 30;6:e29319. doi: 10.7554/eLife.29319 (PMC5662284; doi:10.7554/eLife.29319)
Supplement: Supplementary file 1. [file elife-29319-supp1.docx]

**1. Thematic coding framework for the first phase of interviews conducted (21 researchers at the Neuro)**

*Substantive Categories*

These represent the set of analytical coding categories capturing the substantive material of the interviews: the interviewees’ opinions, experiences and issues relevant to an OSI at the Neuro. This large category includes a subset we derived, called ‘key categories’ (see below for details)

| CATEGORY | NUMBER OF INTERVIEWEES | PERCENTAGE |
| --- | --- | --- |
| *Attribution:* | | |
| Attribution is important for research program and funding | 7 | 33.3% |
| Traditional authorship standards persist for sharing research results and resources | 13 | 61.9% |
| *Changing paradigms in academic research and science in general:* | | |
| Neuro policy (ie. before adoption of Open Science) is lagging behind modern reality of openness | 7 | 33.3% |
| Need for transitional period is MNI moves to Open Science | 4 | 19.0% |
| *Collaborations:* | | |
| Require possibility of patenting discovery | 9 | 42.9% |
| Require secrecy about data generated | 15 | 71.4% |
| Patents are needed to allow collaborations with industry to allow research translation | 9 | 42.9% |
| *Commercialization of research output:* | | |
| Researcher has experience with commercialization | 8 | 38.1% |
| Researchers’ lack of interest in commercializing their research themselves | 10 | 47.6% |
| Increased pressure in recent years felt by researchers to commercialize their research | 7 | 33.3% |
| Commercial entities would take advantage of openness and commercialize research outputs without permission or collaboration, to the detriment of researchers | 6 | 28.6% |
| *Disincentives to sharing or open science:* | | |
| Commercial entities would take advantage of openness | 6 | 28.6% |
| Concerns around privacy and confidentiality of participants | 12 | 57.1% |
| Currently insufficient infrastructure for sharing | 11 | 52.4% |
| Lack of interoperability between potential sharers | 6 | 28.6% |
| Issues around Research Ethics Board (REB) approvals | 3 | 14.3% |
| Ethics approval processes as a barrier | 7 | 33.3% |
| Lack of understanding of REB approval processes | 4 | 19.0% |
| Positive aspects of MNI REB involvement in move towards OS | 12 | 57.1% |
| MNI’s Open Science Initiative, conflict with McGill University patent policy | 2 | 9.5% |
| Losing competitive advantage by sharing too much | 16 | 76.2% |
| Non-issue | 12 | 57.1% |
| Losing out on financial rewards for discovery | 6 | 28.6% |
| Losing private investment in research | 10 | 47.6% |
| Non-issue | 8 | 38.1% |
| Pre-publication sharing/sharing of unvalidated findings may have adverse social impact through poor quality data and resulting research | 10 | 47.6% |
| Researcher time and money invested in resource creation, do not wish to lose through sharing | 10 | 47.6% |
| Time and cost burden of distributing shared resources | 20 | 95.2% |
| Risk of shared data being used out of context/misused and wasting other researchers’ time | 9 | 42.9% |
| *Effects of an OSI on public funding received by the Neuro:* | | |
| Public funding will increase | 7 | 33.3% |
| Public funding will decrease | 6 | 28.6% |
| *Expressed positive perspective on open science* | 15 | 71.4% |
| *Governance mechanisms currently in place or seen as necessary in the context of* Neuro’s Open Science Initiative: |  |  |
| Decisions around data sharing | 17 | 81.0% |
| Decisions around IP | 9 | 42.9% |
| Decisions around use and sharing of valuable resources | 4 | 19.0% |
| Decisions around use and sharing of depletable resources | 3 | 14.3% |
| *Intellectual property (IP):* | | |
| Use others’ patented research tools in their research | 10 | 47.6% |
| Use of others’ patented research tools is not required for their research | 7 | 33.3% |
| Researcher uses IP to protect their research (unspecified) | 1 | 4.8% |
| Copyright | 3 | 14.3% |
| Licenses | 4 | 19.0% |
| Patents | 5 | 23.8% |
| *Motivations for sharing or open science:* | | |
| Existing academic and ethical sharing ethos | 13 | 61.9% |
| Neuro’s Open Science Initiative would attract new students and trainees | 3 | 14.3% |
| Efficient collective problem solving | 19 | 90.5% |
| Increase research impact and dissemination | 12 | 57.1% |
| Increased quality of care and research/ accelerate new cures | 11 | 52.4% |
| Increased stature of Neuro | 6 | 28.6% |
| More and different collaborations | 16 | 76.2% |
| OS leads to more publications | 7 | 33.3% |
| Sharing is the “right and natural” thing to do | 2 | 9.5% |
| Return output of publicly funded research and public resources to the public/ samples/data belong to patients | 4 | 19.0% |
| *Motivations to collaborate with the Neuro:* | | |
| Unique expertise or knowledge | 9 | 42.9% |
| Comprehensiveness of integrated datasets | 5 | 23.8% |
| *Patient-public interest in open science:* | | |
| Interactions between Neuro’s Open Science Initiative and consent | 9 | 42.9% |
| Decreased ease of consent under open science | 3 | 14.3% |
| Increased ease of consent under open science | 3 | 14.3% |
| Public interest in OS is high | 5 | 23.8% |
| Public interest in OS is low | 3 | 14.3% |
| Potential for public trust of the MNI to increase | 5 | 23.8% |
| Potential public trust of the MNI to decrease | 3 | 14.3% |
| *Researchers’ Opinions on IP* | | |
| IP provides advantageous royalty fees | 5 | 23.8% |
| Holding IP protections is a valuable addition to a CV | 5 | 23.8% |
| Distaste for ownership as a public researcher | 5 | 23.8% |
| Excellence in research is more important than patents | 4 | 19.0% |
| Excessive red tape involved in seeking IP | 11 | 52.4% |
| IP is essential to encourage private investment in research | 8 | 38.1% |
| IP protections have negligible value/ rarely worth the investment to obtain | 11 | 52.4% |
| Uncertainty about the value of IP protections | 6 | 28.6% |
| *Solutions:* | | |
| Communication and engagement | 14 | 66.7% |
| Access to shared resources should be controlled | 9 | 42.9% |
| Education of stakeholders (patients and public or researchers) | 13 | 61.9% |
| Need for flexibility or ability to opt-out of Neuro’s Open Science Initiative |  |  |
| Academic freedom or principal investigator/researcher personal freedom to choose | 9 | 42.9% |
| Mandatory open science policy is a bad idea | 11 | 52.4% |
| Need for different models of engagement | 12 | 57.1% |
| Importance of clear goals for Neuro’s Open Science Initiative | 10 | 47.6% |
| Infrastructure support for sharing |  |  |
| Biobanks and data repositories needed | 12 | 57.1% |
| Bioinformaticians and support staff needed | 12 | 57.1% |
| Creation of a patient web-interface needed | 7 | 33.3% |
| Negative opinion | 4 | 19.0% |
| Need for a metric or incentive or credit for openness | 10 | 47.6% |
| Partnership with Research Ethics Board (REB) in Neuro’s Open Science Initiative development and implementation | 4 | 19.0% |
| Publication in open access journals is important to drive openness | 5 | 23.8% |
| Reasonable costs charged for open provision of resources | 3 | 14.3% |
| *Timeline for sharing:* | | |
| Pre-publication sharing of research associated resources is appropriate in some cases; eg. consented genomic data, consented patient data | 16 | 76.2% |
| Post-publication sharing of research associated resources is appropriate in some cases; eg. High resource and intellectual investment outputs | 13 | 61.9% |

*Auxiliary Categories*

Auxiliary categories represent the analytical coding categories that we used to cross-reference interviewees’ opinions, experiences and issues. They include for example, information about the area of research, the reagent, the data-type or the stakeholders the interviewee was speaking about. For example, using these categories in our analysis allowed us to compare how interviewees’ opinions about IP protections differed across types of data or with respect to different stakeholders.

| *Experiences with sharing:* | | |
| --- | --- | --- |
| Data sharing | 16 | 76.2% |
| Not yet sharing but willing | 3 | 14.3% |
| Protocols | 4 | 19.0% |
| Reagents | 9 | 42.9% |
| Only post-publication | 1 | 4.8% |
| Software, algorithms | 5 | 23.8% |
| *Intellectual property:* |  |  |
| Researcher doesn’t use IP | 15 | 71.4% |
| *Past (every mention of anything in the past)* | 14 | 66.7% |
| *Stakeholders:* | | |
| Foundations and private donors | 10 | 47.6% |
| Journals | 9 | 42.9% |
| McGill University | 10 | 47.6% |
| Neuro | 16 | 76.2% |
| Clinical Research Unit | 3 | 14.3% |
| Neurobiobank | 13 | 61.9% |
| Private investors | 17 | 81.0% |
| Biotechnology | 1 | 4.8% |
| Pharmaceutical | 13 | 61.9% |
| Start-ups | 2 | 9.5% |
| Public collaborators | 16 | 76.2% |
| Public funding agencies | 15 | 71.4% |
| CIHR | 10 | 47.6% |
| FRQS | 1 | 4.8% |
| NIH | 6 | 28.6% |
| Public or patients | 14 | 66.7% |
| Public repositories | 13 | 61.9% |
| Addgene | 7 | 33.3% |
| Biobanks | 4 | 19.0% |
| EEG | 0 | 0.0% |
| Fly Bank | 1 | 4.8% |
| Jackson Labs | 4 | 19.0% |
| Parkinson’s Biobank | 1 | 4.8% |
| PPMI | 1 | 4.8% |
| Quebec Parkinson’s Registry | 2 | 9.5% |
| Research Ethics Board (REB) | 10 | 47.6% |
| Structural Genomics Consortium (SGC) | 4 | 19.0% |
| Aled Edwards | 7 | 33.3% |
| SGC model | 3 | 14.3% |
| Uncertainty about merit | 0 | 0.0% |
| Unsure about usefulness | 1 | 4.8% |
| Spin-off companies | 6 | 28.6% |
| *Types of resources:* | | |
| Data | 20 | 95.2% |
| Brain imaging data | 12 | 57.1% |
| Experimental data | 8 | 38.1% |
| Negative data | 4 | 19.0% |
| Genetic data | 11 | 52.4% |
| Patient clinical data | 8 | 38.1% |
| Proteomics datasets | 2 | 9.5% |
| Knowledge | 5 | 23.8% |
| Expertise | 0 | 0.0% |
| Methodologies, protocols, task batteries | 8 | 38.1% |
| Tacit | 0 | 0.0% |
| Reagents | 14 | 66.7% |
| Animal models | 5 | 23.8% |
| Antibodies | 3 | 14.3% |
| Assays | 4 | 19.0% |
| Compounds | 13 | 61.9% |
| iPS cell and other cell-lines | 8 | 38.1% |
| Plasmids | 5 | 23.8% |
| Software and technology | 9 | 42.9% |
| Algorithms | 6 | 28.6% |
| Products and platforms | 5 | 23.8% |
| Tissue samples | 8 | 38.1% |

*Key Categories*

Key categories represent the subset of interviewees’ opinions, concerns and motivations that we determined are the most relevant to the development of an OS policy at the Neuro. Often, this material represents the most significant sources of disagreement or tension about the proposed shift to OS, and material that was the most emphasized by interviewees.

| *Collaborations:* | | | |
| --- | --- | --- | --- |
| Require possibility of patenting discovery | 9 | | 42.9% |
| Collaborations with industry needed to fund research translation | 9 | | 42.9% |
| *Disincentive to sharing or OS:* | | | |
| Commercial entities would take advantage of openness | | 6 | 28.6% |
| Concerns around privacy and confidentiality of participants | | 12 | 57.1% |
| Currently insufficient infrastructure for sharing | | 11 | 52.4% |
| Lack of interoperability between potential sharers (data formatting prevent some users from making use of shared data) | | 6 | 28.6% |
| Issues around Research Ethics Board (REB) approvals | | 3 | 14.3% |
| Ethics approval processes as a barrier | | 7 | 33.3% |
| Lack of understanding of REB processes | | 4 | 19.0% |
| Positive aspects of REB involvement in Open Science | | 12 | 57.1% |
| Losing competitive advantage by sharing too much | | 16 | 76.2% |
| Non-issue | | 12 | 57.1% |
| Losing out on financial rewards for discovery | | 6 | 28.6% |
| Losing private investment in research | | 10 | 47.6% |
| Non-issue | | 8 | 38.1% |
| Pre-publication sharing/ sharing of unvalidated findings may have adverse social impact through poor quality data and resulting research | | 10 | 47.6% |
| Researcher time and money invested in resource creation, do not wish to lose through sharing | | 10 | 47.6% |
| Time and cost burden of distributing shared resources | | 20 | 95.2% |
| Risk of shared data being used out of context/misused and wasting other researchers’ time | | 9 | 42.9% |
| *Governance mechanisms currently in place or seen as necessary in the context of* Neuro’s Open Science Initiative*:* | | | |
| Decisions around data sharing | | 17 | 81.0% |
| Decisions around IP | | 9 | 42.9% |
| *Researcher opinion on IP:* | | | |
| IP provides advantageous royalty fees | | 5 | 23.8% |
| Holding IP protections is a valuable addition to a CV | | 5 | 23.8% |
| Distaste for ownership as a public researcher | | 5 | 23.8% |
| Excellence in research more important than patents | | 4 | 19.0% |
| Excessive red tape involved in seeking IP, or IP itself is excessive red tape | | 11 | 52.4% |
| IP is essential to encourage private investment in research | | 8 | 38.1% |
| IP protections have negligible value | | 11 | 52.4% |
| Uncertainty about the value of patents | | 2 | 9.5% |
| *Motivations for sharing or open science:* | | | |
| Existing academic and ethical sharing ethos | | 13 | 61.9% |
| Efficient collective problem solving | | 19 | 90.5% |
| Increase research impact and dissemination | | 12 | 57.1% |
| Increased quality of care and research /accelerate new cures | | 11 | 52.4% |
| Increased stature of Neuro | | 6 | 28.6% |
| More and different collaborations | | 16 | 76.2% |
| OS leads to more publications | | 7 | 33.3% |
| Return output of publicly funded research and public resources to the public/ samples/data belong to patients | | 4 | 19.0% |
| *Solutions:* | | | |
| Communication and engagement | | 14 | 66.7% |
| Need for flexibility or ability to opt-out of Neuro’s Open Science Initiative | |  |  |
| Academic freedom or principal investigator/researcher personal freedom to choose | | 9 | 42.9% |
| Mandatory open access policy is a bad idea | | 11 | 52.4% |
| Need for different models of engagement | | 12 | 57.1% |
| Importance of clear goals for Neuro’s Open Science Initiative | | 10 | 47.6% |
| Infrastructure support for sharing | | 9 | 42.9% |
| Need for metric or incentive or credit for openness | | 10 | 47.6% |
| *Timeline for sharing:* | | | |
| Pre-publication sharing of research associated resources is appropriate in some cases; eg. consented genomic data, consented patient data | | 16 | 76.2% |
| Post-publication sharing of research associated resources is appropriate in some cases; eg. High resource and intellectual investment outputs | | 13 | 61.9% |
| *Expressed positive perspective on open science* | | 15 | 71.4% |

**2. Thematic coding framework for the second phase of interviews** (four MNI researchers (three re-interviews of researchers interviewed in the first phase of the study; one new interviewee) and three collaborators/industry stakeholders as described in Methods: study sample: one industry collaborator, one industry veteran and one not-for-profit collaborator)

| CATEGORY | MNI RESEARCHERS | INDUSTRY COLLABORATORS | INDUSTRY VETERANS | NON-PROFIT COLLABORATORS |
| --- | --- | --- | --- | --- |
| *Attitudes of industry toward collaboration with the MNI under open science principles* | | | | |
| Positive or curious towards collaboration under open science requirement for public data release; easier access to scientific outputs/biobank materials | 4/4 | 1/1 | 1/1 | 1/1 |
| Negative or wary toward collaboration under open science; requirement for public data release | 1/4 | 0/1 | 0/1 | 1/1 |
| MNI will not insist on release of industry data in clinical trials context | 1/4 | 0/1 | 0/1 | 0/1 |
| Negative or wary toward collaboration under open science; concern about legal implications of sharing of encumbered materials | 0/4 | 1/1 | 0/1 | 1/1 |
